# Effects of Sophorolipid on Growth Performance, Organ Characteristics, Lipid Digestion Markers, and Gut Functionality and Integrity in Broiler Chickens

**DOI:** 10.3390/ani12050635

**Published:** 2022-03-02

**Authors:** Min-Jin Kwak, Sun-Woo Choi, Yong-Soon Choi, Hanbae Lee, Min-Young Park, Kwang-Youn Whang

**Affiliations:** 1 Department of Biotechnology, Korea University, Seoul 02841, Korea; ics1051@korea.ac.kr (M.-J.K.); swchoi@ctcbio.com (S.-W.C.); dj1235@korea.ac.kr (Y.-S.C.); bennjerrry@hotmail.com (M.-Y.P.); 2 Division of Interdisciplinary Program in Precision Public Health (BK21 FOUR Program), Department of Biomedical Engineering, Korea University, Seoul 02841, Korea; 3 Pathway Intermediates, Seoul 02841, Korea; hanbae.lee@pathway-intermediates.com

**Keywords:** broiler chickens, sophorolipid, gut microenvironments, lipid bioavailability, growth performance

## Abstract

**Simple Summary:**

Availability of dietary fat and oil is important to broiler chicken due to their rapid growth rate. Therefore, we conducted an experiment with dietary sophorolipid, a glycolipid-type emulsifier, to investigate growth, lipid digestion markers and gut health during the growing period. Growth was accelerated by dietary sophorolipid supplementation through upregulation of lipid digestion and absorption markers. Additionally, dietary sophorolipid also increased the surface area of the gut and modulated microbial population and short-chain fatty acid concentration. Collectively, this study proposed that sophorolipid addition in feed could enhance chicken’s growth by increased intestinal absorption of dietary lipid and improved gut microenvironments.

**Abstract:**

Dietary fat and oil could aid in reaching the high-energy requirements of fast-growing birds; however, these inclusions could lead to nutrient waste. This is because young birds have limited lipid digestion due to the low secretion of lipase and bile salt. Sophorolipid (SPL), a glycolipid emulsifier with lower toxicity and higher biodegradability, can upregulate fat utilization by increasing digestibility. Accordingly, a five-week-long experiment was conducted with 720 one-day-old chicks (Ross 308) to investigate the effects of dietary SPL on growth, organ characteristics, and gut health. The allotment was partitioned into four treatment groups according to their body weight with six replications (30 chick/pen). The three treatment diets comprised a basal diet with a formulation that met the Ross 308 standard and 5, 10, and 15 ppm SPL in the basal diet. During the experiment, the birds had free access to feed, and body weight and feed intake were measured at the end of each phase. Chickens were put down at the end of the growing and finishing phases, and jejunum and cecal samples were obtained to investigate organ characteristics and gut environments. The data were analyzed using the generalized linear model procedures of SAS 9.4, and all data were assessed for linear, quadratic, and cubic effects of dietary SPL-supplemented dosages. Body weight was significantly increased with 10 ppm of SPL supplementation in the grower phase without affecting feed efficiency. The relative weights of the intestine and the bursa of Fabricius were quadratically decreased by SPL supplementation with a lower population of *Streptococcus* and higher propionate and butyrate concentrations. Additionally, the dietary SPL supplementation groups showed a significantly increased villus/crypt ratio with higher intestinal expression levels of fatty acid translocase, diacylglycerol acyltransferase 2, and fatty acid transporter 4. Collectively, proper SPL supplementation in the chicken diet could improve growth performance by down-regulating immune modulation and up-regulating lipid digestion and absorption via modulation of gut microenvironments.

## 1. Introduction

Fat and oil inclusions in the feed formulation of young birds can help meet the high-energy requirements of fast-growing birds [1]. Dietary inclusion of fat could improve feed efficiency and carcass quality by supplying essential fatty acids and vitamins and lowering the passage rate in the gastrointestinal tract [2,3]. However, feeding young birds with high quantities of lipids remains a controversial issue for the livestock industry. One reason is that the supplementation of fat in feed formulation leads to a dramatic increase in feed price, with another being that young broiler chicks have physiological hurdles in lipid digestion due to the low secretion of lipase and bile salts [4,5]. In 2020, Mohamed et al. demonstrated that 0.5 and 1.0 mL/kg of dietary bile salt supplementation could improve growth performance, blood biochemical markers, gut enzyme activities, digestibility of nutrients, and microbial population [6]. However, dietary single enzyme supplementation is hard to maximize the lipid digestibility, due to the complex fat digestion process, as it includes breakdown of the fat droplet, emulsification, lipolysis, and micelle formation [7]. 

Therefore, various studies have also been investigating the effects of exogenous lipases in the poultry and livestock industry. Lipase is an endogenous enzyme in the digestive tract, and it hydrolyzes absorbed triglyceride to glycerol and free fatty acid. In 2018, Hu et al. demonstrated that dietary 300 U/kg of lipase supplementation could increase feed efficiency, digestibility, and decrease the concentration of serum lipid markers, and the abdominal fat portion in broilers [8]. However, since most lipases cannot tolerate high temperature, pH, and organic solvent, various researchers have been seeking to improve the stability and utilization of exogenous enzyme preparation [9]. One of the strategies to replace in exogenous enzymes is to maximize the effects of existing lipase in broiler’s intestine by emulsifying dietary fat. Various emulsifiers have been reported to improve growth performance and nutrient digestion in broiler chickens; however, the toxicity and biodegradability of emulsifier have raised concerns in public health [10,11]. In this situation, we suggested an exogenous surfactant, sophorolipid (SPL), as a novel dietary additive in broiler industry. 

SPL, a type of glycolipid biosurfactant produced by *Candida bombicola*, comprises a nonpolar fatty acid and a polar dimeric carbohydrate head linked by a glycosidic bond [12,13]. Diverse SPL research has demonstrated that SPL is a novel and eco-friendly surfactant with lower toxicity and higher degradability [14]. Additionally, they also proposed that SPL could exert various unique properties, including immune modulation, fibroblast stimulation, and collagen neogenesis [15,16]. These SPL properties imply that it could be applied in various industrial areas, such as medical, hygiene, pharmacodermatological, and domestic fields. In 2021, SPL was evaluated in the animal feed industry; however, the lipid digestion capacity of SPL has not been investigated [17]. Herein, an experiment with broiler chickens was conducted to investigate dietary effects of SPL on growth performance, organ characteristics, lipid digestion and absorption markers, and gut microenvironments. 

## 2. Materials and Methods

### 2.1. Animals, Diets, and Housing

All of works related to chicks were conducted in accordance with the guidelines of the Animal Ethics Committee approved by the Korea University, South Korea (KU-2020-0082). This study was conducted during 35 days at a research farm in Cheonan, Republic of Korea. We used 720 one-day-old male chicks (Ross 308) with an average body weight of 40.14 ± 0.12 g, which were randomly allotted to four experimental treatment groups according to their initial BW with six replication (30 chicks/pen). The basal diets consisted of three phases: starter phase (day 0–10), grower phase (day 11–20), and finisher phase (day 21–35). Dietary SPL was supplemented at three dosages (5, 10, and 15 ppm). We had prepared three dosages of SPL supplemented premixes equivalent to 10% of total weight of feed, and the premix was mixed with the total amount of feed. Table 1 presents the formulation and nutrient specification of the basal diet, and feed and SPL were supported by EASY BIO Inc. (Seoul, South Korea). Chicks were raised in an environmentally controlled room with rice hulls. Feed and water were freely provided to the chicks, and the lighting program provided artificial light for 24 h/d.

### 2.2. Experimental Procedures and Sample Collection

The body weight by pen and feed intake of birds were recorded by pen at the end of each period after 8 h of feed deprivation. The data were used to calculate the average daily gain (ADG), average daily feed intake (ADFI), and feed conversion ratio (FCR) for each period and throughout the experiment. At the end of the grower (day 20) and finisher periods (day 35), 48 chicks (24 on the final day of the grower phase and the others on the final day of the finisher phase, one bird per pen) were sacrificed and the relative weights of the intestine, spleen, and the bursa of Fabricius were recorded. Jejunal and cecal content samples were obtained. Cecal contents samples and a part of the jejunum samples were immediately frozen by liquid nitrogen and stored in a deep freezer (−80 °C), and the other part of the jejunum samples was fixed with 4% formalin solution. 

### 2.3. Morphological Assay

All the fixed jejunum samples were embedded in paraffin for 5-μm section preparation using a rotary microtome CUT 5062 (SLEE MAINZ, Mainz, Germany). The jejunum sections were stained with hematoxylin and eosin to measure villus height and crypt depth, and Alcian blue staining was carried out to determine the number of goblet cells.

### 2.4. RNA and Microbial DNA Extraction 

Total RNA content from the jejunum samples was extracted using TRIzol^®^ (Invitrogen, Grand Island, NY, USA) and extracted RNA was evaluated by a Nanodrop spectrophotometer (Thermo Scientific, Wilmington, DE, USA). cDNA samples were immediately synthesized with a High-Capacity cDNA Reverse Transcription kit (Applied Biosystems, Foster City, CA, USA). The total genomic DNA content of chicken’s feces was extracted using the PowerSoil^®^ DNA Isolation Kit (Mo BIO Laboratories, Carlsbad, CA, USA) according to the manufacturer’s protocol.

### 2.5. qRT-PCR

The jejunal gene expression levels of lipid absorption proteins (fatty acid binding protein 1, FABP1; fatty acid translocase, CD36; diacylglycerol acyltransferase 2, DGAT2; fatty acid transporter 4, FATP4) were determined by a StepOnePlus Real-Time PCR System (Applied Biosystems) with a RealHelixTM Premier qPCR kit (NanoHelix, Daejeon, Korea). Glyceraldehyde-3-phosphate dehydrogenase (GAPDH) was used as a housekeeping gene. In addition, specific microbial populations (*E. coli*, *Streptococcus* spp., and *Salmonella* spp.) were determined, and total bacteria were used as housekeeping bacteria. The 2^−ΔΔCT^ method was used to quantify the relative mRNA expression levels. The primers used for the target genes are listed in Table 2.

### 2.6. Short-Chain Fatty Acid Measurement

The concentration of short-chain fatty acids (SCFAs) in the cecal contents was determined using gas chromatography-mass spectrometry (GC-MS) according to the method of Furuwasa et al. [25]. 

### 2.7. Statistical Analysis

Data were analyzed using the GLM procedure in SAS software (version 9.4; SAS Institute, Cary, NC, USA). Significant differences between treatment groups were determined by Duncan’s multiple-range tests, and they were divided at the *p* < 0.05 level. The experimental unit of growth performance was a pen, and one randomly selected bird from each pen was defined as the experimental unit for relative organ weights, serum biochemical markers, morphological measurements, and gene expression levels. All data were assessed for linear, quadratic, and cubic effects of dietary dosages of SPL supplementation.

## 3. Results

### 3.1. Growth Performance

The effects of SPL supplementation on the growth performance of birds during the five-week experimental period are presented in Table 3. At the end of the grower phase, 10 ppm of SPL supplementation could accelerate the body weight of broiler chickens compared to 15 ppm of SPL supplementation (*p* < 0.05). Additionally, ADFI in the grower phase was quadratically increased by SPL inclusion dosages (*p* < 0.05). However, FCR was not affected by dietary SPL supplementation.

### 3.2. Relative Organ Weights

The relative organ weights of broiler chicks fed the experimental diets during the growing and finishing phases are summarized in Table 4. On day 20, gut weight per length was significantly decreased by 5 and 10 ppm of SPL supplementation, and it showed quadratic relationship according to SPL dosages (*p* < 0.05). Furthermore, the relative weights of the spleen and the bursa of Fabricius were also significantly decreased by the addition of 10 ppm SPL. A quadratic relationship was observed in 5 and 10 ppm supplemented groups at day 35 (*p* < 0.05). 

### 3.3. Morphology of Small Intestine

**Figure 1:**
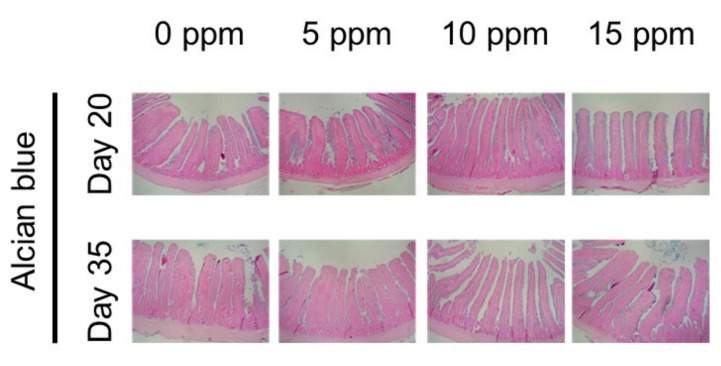
Representative pictures of jejunum stained with Alcian blue staining methods.

The dietary effects of SPL supplementation on morphological indices of the small intestine are listed in Table 5, and the representative picture of intestine is shown in Figure 1. On day 20, villus heights in the 10-ppm supplemented treatment was significantly higher, and crypt depths in all of SPL supplemented groups were significantly lower compared to the 0-ppm group (*p* < 0.05). Dietary SPL supplementation significantly increased villus crypt ratio and goblet cell population (*p* < 0.05), and a linear and quadratic relationship was found in all SPL supplemented groups in all morphological indices (*p* < 0.05). Similarly, higher villus height and villus crypt ratio were found in the 10- and 15-ppm of SPL supplemented groups with a linear relationship (*p* < 0.05).

### 3.4. Gene Expression Levels of Lipid Digestion Markers in Jejunum

Lipid digestion markers (FABP1, CD36, FATP4, and DGAT2) in the jejunum of broiler chickens fed experimental diets are shown in Table 6. The expression levels of CD36 on day 20 and 35 were significantly higher in the 15-ppm SPL supplemented group with linear increasing tendency by SPL inclusion dosage levels (*p* < 0.05). All of the SPL supplemented group showed a significantly lower expression level in FATP4 on day 20, and a linear decrease was observed in all SPL supplemented groups (*p* < 0.05). The expression level of DGAT2 was significantly higher in the 10- and 15-ppm of SPL supplemented groups on day 35 with linear increasing tendency (*p* < 0.05). 

### 3.5. Microbial Population in Cecal Contents

The microbial populations (*E. coli*, *Streptococcus* spp., and *Salmonella* spp.) in the cecal contents of broiler chicks fed experimental diets during the growth and finishing phases are summarized in Table 7. The populations of *E. coli* and *Salmonella* were not changed by SPL supplementation on both day 20 and 35. However, dietary SPL addition could significantly reduce a population of *Streptococcus* spp. on day 20, and it showed a linear relationship with SPL dosage (*p* < 0.05).

### 3.6. Short Chain Fatty Acid Concentration in Cecal Contents

**Figure 2:**
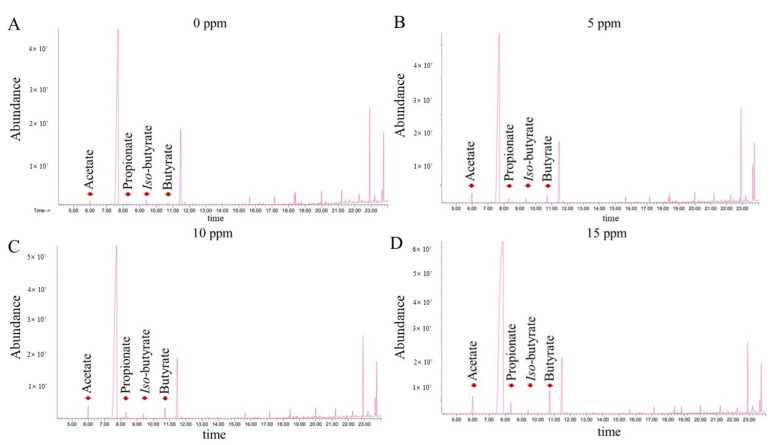
GC-MS analysis of representative fraction on day 35. Acetate: 5.00 min; propionate: 8.00 min; *iso*-butyrate: 9.45 min; butyrate: 10.75 min. (**A**–**D**) GC-MS representative fraction of chickens fed 0, 5, 10, 15 ppm of SPL supplemented diet.

The SCFA concentrations in the cecum of broiler chicks fed the experimental diets during the growing and finishing phases are listed in Table 8, and the representative fraction of GC-MS analysis on day 35 is shown in Figure 2. The concentration of butyrate was significantly increased by 5 and 10 ppm of SPL supplementation on day 20, and it was quadratically increased by SPL inclusion dosages (*p* < 0.05). On day 35, propionate concentration was significantly increased by 10 and 15 ppm of SPL addition with linear increasing tendency (*p* < 0.05). 

## 4. Discussion

Dietary SPL supplementation has previously been investigated in broiler chickens, and recent results demonstrate that SPL addition in feed could positively boost growth performance by improving the gut microenvironment, including the microbiota population and defense systems [17]. Consistent with previous studies, our results indicated that dietary SPL supplementation could increase chick’s growth during the grower phase. Khonyoung et al. also proposed that supplementation with dietary emulsifiers could improve the FCR of young chicks by upregulating the lipid digestion of feed [26]. In addition, dietary emulsifiers can improve growth performance during the entire chicken lifespan without affecting feed intake [27]. Collectively, an adequate dosage of SPL could accelerate the growth of chickens by fortifying the gut barrier function and promoting lipid digestion and absorption.

In addition, the bursa of Fabricius is a major immune organ in chickens that produces antibodies against systemic immune responses [28]. The results of this study suggest that dietary SPL supplementation ameliorates systemic immune response; however, further studies will be needed. Bontempo et al. demonstrated that dietary synthetic emulsifier supplementation could ameliorate inflammation in broiler chickens by modulating the gut microbiome, including lactobacilli and *E. coli* [29]. In accordance with this study, our research also suggests that this might be due to fortified gut barrier functions, including mucus barrier and morphological indexes, and antimicrobial effects on the gut pathogenic bacterial population of *Streptococcus* species.

Various lipid digestion and absorption markers (FABP1, CD36, FATP4, and DGAT2) were investigated to elucidate the mode of action of SPL in the emulsifying effect of fat substrates in feed. FABP1 is a soluble molecule in the intestinal mucosa, and it can bind to fatty acids to change dietary fat into a smooth form for absorption through cellular fatty acid transport, including FATP4 [30]. Consistent with our study, Huang et al. suggested that dietary soy lecithin, an emulsifier, could increase lipid absorption by increasing the gene expression levels of FATP4, but not FABP1 [29,31]. CD36 is a protein that transports long-chain fatty acids and was found to have a positive impact on long-chain fatty acid uptake capacity, while DGAT2 plays a vital role in fat metabolism and lipid deposition in chickens [32,33,34]. Our results indicated that dietary SPL could increase fatty acid digestion and absorption. This might be due to the direct regulatory role of SPL on enterocytes; however, further studies on the relationship between the beneficial gut microbiome and lipid metabolism are needed.

It is widely known that cecal SCFA production by bacterial fermentation is necessary for intestinal functionality and integrity [35]. In particular, acetate and propionate concentrations in the cecum could have antimicrobial effects on pathogenic bacteria [36]. Moreover, butyrate, which can be used as an energy source for enterocytes, enhances feed efficiency by upregulating nutrient transport. [37,38]. In our study, dietary SPL supplementation quadratically increased SCFA production at high concentrations of propionate and butyrate. Our previous studies also showed that SPL-supplemented feed could enhance SCFA concentrations by modulating the gut microbiota population in diverse animal models [39,40,41].

## 5. Conclusions

Collectively, this study proposed that dietary SPL supplementation at a dosage of 10 ppm could be beneficial to growth performance by decreasing systemic and local inflammation and improving lipid digestion and absorption via a lower population of pathogenic bacteria and a higher concentration of SCFA production.

## Figures and Tables

**Table 1:** Composition and nutrient specification of the basal diets (%).

	Starter (day 0–10)	Grower (day 11–20)	Finisher (day 21–35)
Ingredients			
Corn	54.23	49.10	55.95
Soybean meal	30.38	22.03	14.05
Fermented soybean meal	5.00	0.00	0.00
Distilled dried grains with solubles	0.00	5.00	5.00
Unpolished rice	0.00	4.00	3.00
Rice bran polished	0.00	1.00	1.50
Rapeseed mineral	0.00	4.00	3.00
Sesame seed meal	0.00	0.00	0.50
Poultry meal	2.50	5.50	8.00
Animal fat	2.47	5.41	5.47
Soy oil	0.90	0.00	0.00
L-Lysine sulfate (55%)	0.46	0.57	0.62
L-Methionine (90%)	0.45	0.32	0.29
Threonine (98%)	0.17	0.14	0.15
L-Tryptophan (99%)	0.00	0.01	0.02
Choline chloride (50%)	0.10	0.10	0.12
Monocalcium phosphate	1.53	1.07	0.80
Limestone	1.18	1.20	1.00
Salt	0.25	0.25	0.25
Sodium bicarbonate	0.05	0.05	0.05
Vitamin premix ^1^	0.20	0.14	0.11
Mineral premix ^2^	0.15	0.12	0.12
Total	100.00	100.00	100.00
Calculated value			
Metabolizable energy (kcal/kg)	3002.00	3100.00	3200.00
Crude protein (%)	23.00	21.50	20.00
Crude fat (%)	5.96	8.63	9.39
Calcium (%)	0.90	0.90	0.85
Phosphate (%)	0.77	0.71	0.65
Lysine (%)	1.50	1.33	1.20
Methionine (%)	0.74	0.61	0.56
Threonine (%)	1.03	0.95	0.90
Tryptophan (%)	0.26	0.23	0.20

^1^ Provided per kilogram of complete diet: vitamin A, 6300 IU; vitamin D, 2800 IU; vitamin E, 35 mg; vitamin K_3_, 1.75 mg; vitamin B_1_, 2 mg; vitamin B_2_, 6 mg; vitamin B_6_, 3 mg; vitamin B_12_, 13 μg; biotin, 0.1 mg; calcium pantothenic acid, 15 mg; folic acid, 1.5 mg; niacin, 50 mg. ^2^ Provided per kilogram of complete diet: Mn, 100 mg; Cu, 17 mg; Zn, 92 mg; Fe, 50 mg; I, 1.5 mg; Co, 0.15 mg; Se 0.3 mg.

**Table 2:** Oligonucleotide primers used in qRT-PCR analysis ^1^.

Gene Name	Sequence (Forward, Reverse)	Reference
Housekeeping gene		
GAPDH	F: 5′-CTACACACGGACACTTCAAG-3′	[18]
	R: 5′-GACTACGGGGGTACAAACA-3′	
Lipid absorption proteins		
FABP1	F: 5′-ACTGGCTCCAAAGAATGACCAATG-3′	[19]
	R: 5′-TGTCTCCGTTGAGTTCGGTCAC-3′	
CD36	F: 5′-GCGATTTGGTTAATGGCACT-3′	Self-made
	R: 5′-TCTCCAACATCAATCGGTGA-3′	
DGAT2	F: 5′-AAAAGGGGATGCTGCCTATCT-3′	[20]
	R: 5′-GCTTACGCAGCTCCATCTTCT-3′	
FATP4	F: 5′-AGGGATTTGTGAAACTGGCACT-3′	[20]
	R: 5′-CTTTGGGATGGTGATGGGTT-3′	
Intestinal microbial species		
Total bacteria	F: 5′-GCAGGCCTAACACATGCAAGTC-3′	[21]
	R: 5′-CTGCTGCCTCCCGTAGGAGT-3′	
*E. coli*	F: 5′-CATGCCGCGTGTATGAAGAA-3′	[22]
	R: 5′-CGGGTAACGTCAATGAGCAAA-3′	
*Sterptococcus* spp.	F: 5′-GTACAGTTGCTTCAGGACGTATC-3′	[23]
	R: 5′-ACGTTCGATTTCATCACGTTG-3′	
*Salmonella* spp.	F: 5′-AACGTGTTTCCGTGCGTAAT-3′	[24]
	R: 5′-TCCATCAAATTAGCGGAGGC-3′	

^1^ Abbreviations: CD36, cluster of differentiation 36; DGAT2, diacylglycerol o-acyltransferase; FABP1, fatty-acid binding protein 1; FATP4, fatty-acid transport protein 4; GAPDH, glyceraldehyde-3-phosphate dehydrogenase.

**Table 3:** Growth performance of broiler chickens fed experimental diets ^1^.

Treatment	0 ppm	5 ppm	10 ppm	15 ppm	SEM	*p*-Value	Linear	Quadratic	Cubic
Body weight, g									
Day 0	40.09	40.14	40.18	40.15	0.029	0.599	0.476	0.257	0.962
Day 10	259.74	256.88	261.32	247.78	3.498	0.083	0.072	0.181	0.175
Day 20	868.27 ^ab^	866.79 ^ab^	878.80 ^a^	841.43 ^b^	5.164	0.043	0.084	0.057	0.141
Day 35	2004.80	2060.65	2047.47	2005.17	13.184	0.338	0.915	0.080	0.747
ADG, g/day									
Starter	21.97	21.67	22.11	20.76	0.216	0.087	0.073	0.188	0.180
Grower	60.85	60.99	61.75	59.37	0.345	0.077	0.173	0.055	0.204
Finisher	75.77	79.59	77.91	77.58	0.779	0.418	0.583	0.206	0.364
Overall	56.13	57.73	57.35	56.14	0.377	0.338	0.914	0.080	0.747
ADFI, g/day									
Starter	27.92	28.58	27.93	27.24	0.313	0.547	0.337	0.311	0.685
Grower	86.40	87.52	90.16	84.91	0.728	0.062	0.739	0.023	0.131
Finisher	134.37	135.98	138.26	133.92	1.474	0.769	0.943	0.362	0.624
Overall	89.33	90.15	91.58	88.39	0.682	0.441	0.823	0.167	0.431
FCR									
Starter	1.27	1.32	1.26	1.31	0.012	0.269	0.494	0.993	0.067
Grower	1.42	1.44	1.46	1.43	0.009	0.488	0.472	0.244	0.451
Finisher	1.77	1.71	1.78	1.73	0.014	0.255	0.529	0.795	0.070
Overall	1.59	1.56	1.60	1.58	0.008	0.546	0.839	0.856	0.170

^1^ Mean values represent six replicates per treatment; pen is the experimental unit. Abbreviations: ADFI, average daily feed intake; ADG, average daily gain; BW, body weight; FE, feed efficiency; SEM, standard error of means. ^a,b^ Values within a row with no common letters differ significantly (*p* < 0.05).

**Table 4:** Relative organ weights in broiler chickens fed experimental diets ^1^.

Treatment	0 ppm	5 ppm	10 ppm	15 ppm	SEM	*p*-Value	Linear	Quadratic	Cubic
Day 20									
Intestine, g/kg	49.35	45.13	46.75	50.21	1.063	0.353	0.658	0.088	0.672
Gut weight/length, g/m	27.52 ^a^	23.85 ^b^	24.54 ^b^	27.34 ^a^	0.664	0.037	0.980	0.016	0.669
Spleen, g/kg	0.79	0.89	0.81	0.85	0.040	0.540	0.474	0.635	0.219
Bursa of Fabricius, g/kg	1.85	1.78	1.86	1.90	0.078	0.838	0.842	0.677	0.446
Day 35									
Intestine, g/kg	31.01	30.83	28.81	30.16	0.816	0.808	0.570	0.669	0.521
Gut weight/length, g/m	31.90	30.34	30.33	29.74	0.787	0.808	0.389	0.770	0.777
Spleen, g/kg	1.42 ^a^	0.80 ^b^	0.75 ^b^	0.93 ^a^	0.082	0.001	0.004	0.001	0.476
Bursa of Fabricius, g/kg	1.13 ^a^	0.92 ^ab^	0.71 ^b^	1.21 ^a^	0.069	0.021	0.946	0.005	0.145

^1^ Mean values represent six replicates per treatment; chick is the experimental unit. ^a,b^ Values within a row with no common letters differ significantly (*p* < 0.05).

**Table 5:** Morphological indexes in jejunum of broiler chicks fed experimental diets ^1^.

Treatment	0 ppm	5 ppm	10 ppm	15 ppm	SEM	*p*-Value	Linear	Quadratic	Cubic
Day 20									
Villus height, μm	316.53 ^c^	335.94 ^b^	403.85 ^a^	364.62 ^ab^	11.173	0.013	0.013	0.096	0.053
Crypt depth, μm	107.78 ^a^	75.99 ^b^	80.22 ^b^	81.79 ^b^	3.734	0.001	0.001	0.002	0.062
Villus crypt ratio	2.95 ^b^	4.42 ^a^	5.04 ^a^	4.48 ^a^	0.213	<0.001	<0.001	<0.001	0.673
Goblet cells/villus, μm	0.21 ^b^	0.36 ^ab^	0.44 ^a^	0.51 ^a^	0.032	<0.001	<0.001	0.205	0.756
Day 35									
Villus height, μm	346.11 ^b^	416.61 ^ab^	435.86 ^a^	449.26 ^a^	14.836	0.044	0.010	0.256	0.679
Crypt depth, μm	103.72	102.16	93.43	87.00	3.183	0.214	0.047	0.690	0.728
Villus crypt ratio	3.34 ^b^	4.09 ^ab^	4.68 ^a^	5.26 ^a^	0.224	0.003	<0.001	0.776	0.907
Goblet cells/villus, μm	0.27	0.32	0.35	0.30	0.016	0.426	0.423	0.175	0.641

^1^ Mean values represent six replicates per treatment; chick is the experimental unit. ^a–c^ Values within a row with no common letters differ significantly (*p* < 0.05).

**Table 6:** Lipid absorption of genes in the jejunum of broiler chicks fed experimental diets ^1,2^.

Treatment	0 ppm	5 ppm	10 ppm	15 ppm	SEM	*p*-Value	Linear	Quadratic	Cubic
Day 20									
FABP1, fold change	1.00	0.99	1.30	3.00	0.383	0.284	0.110	0.370	0.841
CD36, fold change	1.00 ^b^	1.13 ^b^	1.22 ^b^	2.04 ^a^	0.177	0.035	0.036	0.298	0.611
FATP4, fold change	1.00 ^a^	0.57 ^ab^	0.61 ^ab^	0.47 ^b^	0.064	0.029	0.008	0.142	0.197
DGAT2, fold change	1.00	1.58	1.03	1.19	0.150	0.562	0.956	0.543	0.200
Day 35									
FABP1, fold change	1.00	1.33	1.67	1.38	0.199	0.460	0.128	0.850	0.934
CD36, fold change	1.00 ^b^	1.04 ^b^	1.60 ^ab^	3.07 ^a^	0.278	0.048	0.021	0.282	0.998
FATP4, fold change	1.00	0.74	1.52	1.41	0.175	0.140	0.064	0.474	0.255
DGAT2, fold change	1.00 ^b^	0.92 ^b^	2.06 ^ab^	3.52 ^a^	0.381	0.019	0.004	0.176	0.758

^1^ Mean values represent six replicates per treatment; chick is the experimental unit. ^2^ Abbreviations: CD36, fatty acid translocase; DGAT2, diacylglycerol acyltransferase 2; FABP1, fatty acid binding protein 1; FATP4, fatty acid transporter 4. ^a,b^ Values within a row with no common letters differ significantly (*p* < 0.05).

**Table 7:** Specific microbial population in cecum of broiler chicks fed experimental diets ^1^.

Treatment	0 ppm	5 ppm	10 ppm	15 ppm	SEM	*p*-Value	Linear	Quadratic	Cubic
Day 20									
*E. coli,* fold change	1.00	0.46	0.89	0.51	0.164	0.636	0.245	0.930	0.701
*Streptococcus* spp., fold change	1.00 ^a^	0.17 ^b^	0.16 ^b^	0.23 ^b^	0.132	0.035	0.017	0.092	0.327
*Salmonella* spp., fold change	1.00	1.06	1.17	1.10	0.109	0.973	0.912	0.691	0.808
Day 35									
*E. coli,* fold change	1.00	1.64	0.64	1.45	0.184	0.224	0.105	0.435	0.268
*Streptococcus* spp., fold change	1.00	0.32	0.89	0.84	0.144	0.203	0.219	0.253	0.282
*Salmonella* spp., fold change	1.00	0.77	0.45	0.72	0.092	0.256	0.584	0.119	0.257

^1^ Mean values represent six replicates per treatment; chick is the experimental unit. ^a,b^ Values within a row with no common letters differ significantly (*p* < 0.05).

**Table 8:** Short chain fatty acid concentrations in the cecum of broiler chickens fed experimental diets ^1^.

Treatment	0 ppm	5 ppm	10 ppm	15 ppm	SEM	*p*-Value	Linear	Quadratic	Cubic
Day 20									
Acetate, mmol/g	107.99	169.39	148.70	127.02	12.007	0.328	0.732	0.108	0.453
Propionate, mmol/g	8.44	13.95	13.52	14.99	1.410	0.402	0.161	0.489	0.545
*Iso*-butyrate, mmol/g	2.67	3.16	2.55	2.77	0.223	0.839	0.905	0.790	0.412
Butyrate, mmol/g	8.97 ^b^	19.03 ^a^	21.02 ^a^	11.25 ^b^	2.301	0.029	0.637	0.039	0.844
Total, mmol/g	128.06	205.53	185.79	156.04	15.007	0.304	0.627	0.096	0.512
Day 35									
Acetate, mmol/g	149.20	165.49	187.14	206.04	12.292	0.430	0.117	0.959	0.943
Propionate, mmol/g	12.97 ^b^	16.17 ^a^^b^	22.01 ^a^	22.91 ^a^	1.758	0.016	0.026	0.705	0.578
*Iso*-butyrate, mmol/g	3.39	3.96	3.96	4.24	0.330	0.872	0.466	0.844	0.805
Butyrate, mmol/g	15.47	19.68	23.45	25.32	2.494	0.578	0.190	0.829	0.951
Total, mmol/g	181.04	205.30	236.56	258.51	16.284	0.390	0.102	0.972	0.912

^1^ Mean values represent six replicates per treatment; chick is the experimental unit. ^a,b^ Values within a row with no common letters differ significantly (*p* < 0.05).

## Data Availability

Not applicable.
